# Antimicrobial Stewardship Program (ASP) in General Hospital: An Essential Practice

**DOI:** 10.3390/antibiotics13121108

**Published:** 2024-11-21

**Authors:** Michael Samarkos

**Affiliations:** 1st Department of Internal Medicine, Laikon Hospital, Medical School, National and Kapodistrian University of Athens, 115 27 Athens, Greece; msamarkos@med.uoa.gr; Tel.: +30-2132061149

## 1. Introduction

Antimicrobial drugs are unique among the different categories of medications as their inappropriate use has a negative impact both for the individual patient, as is the case with all other medications, and for the general population as well. Thus, inappropriate antimicrobial use is associated with patient-specific adverse events, such as C. difficile infection; however, it also has adverse consequences for the whole population, in the form of the development of antimicrobial resistance and the unnecessary costs of antimicrobials. For these reasons, antimicrobial drugs have been characterized as “societal drugs” [1]. In terms of the value of care, inappropriate use of antimicrobials has been characterized as “perhaps one of the most threatening forms of wasteful clinical care” [2]. Inappropriate and/or unnecessary use of antimicrobials is widespread, across various levels of care and countries, with estimates of inappropriate and/or unnecessary use reaching 50% in the US [3]. It is, therefore, imperative that the use of antimicrobials be optimized, and the tools for this are provided by antimicrobial stewardship.

Antimicrobial stewardship includes interventions designed to improve the use of antimicrobial agents by promoting the selection of the optimal drug regimen, including dosing, duration of therapy, and route of administration. As more than one clinician might be involved in these interventions (e.g., infectious diseases specialists, pharmacists, and clinical microbiology specialists), they should collaborate, and their treatment effects should be monitored [4]. This monitoring should inform a Plan–Do–Study–Act cycle, which in turn might lead to modification of the intervention.

The ultimate goal of antimicrobial stewardship is to improve patient outcomes; thus, this is a “fiduciary responsibility” of the physician and should be applied to any instance of antimicrobial use regardless of the level of care [4]. Although the large volume of antimicrobial prescribing occurs in the community, antimicrobial stewardship programs (ASPs) have mostly been employed in hospitals. The reason for this might be that ASPs include complex interventions and multiple structural and procedural components which are difficult to implement in outpatient settings [5]. In addition, antimicrobial use in hospitals is widespread, with a study including 323 hospitals in the US in 2010 showing that 56% of discharged patients received at least one antibiotic during their hospitalization. Finally, some additional goals of ASPs, such as improving susceptibility rates to targeted antimicrobials and optimizing resource utilization, might be more immediately relevant to hospital settings [6].

The importance of ASPs for the contemporary hospital is underscored by the fact that in the United States, implementation of ASPs is required by the Centers for Disease Control and Prevention (CDC) and Centers for Medicare and Medicaid Services (CMS) in order for health care institutions to receive CMS funding [5]. In addition, Joint Commission accreditation also requires the presence of an ASP [7].

## 2. Overview of the Published Articles

The present Special Issue includes five studies covering various aspects of ASPs in General Hospitals.

Two of the studies address the issue of carbapenem-resistant pathogens. In the first study, López-Viñau et al. report the results of a 6-year mixed ASP intervention targeting carbapenem-resistant Klebsiella pneumoniae (CR-Kp) [8]. The outcomes were carbapenem use and incidence density, as well as crude death rates of hospital-acquired CR-Kp infections. The authors analyzed the data using interrupted time series and found that carbapenem use decreased significantly early post-intervention and stayed rather stable over the long term, reaching a decrease by 66.19% at the end of the study, in comparison with the pre-intervention expected value. It is important to note that consumption of fluoroquinolones decreased, while that of beta-lactams/beta-lactamase inhibitors increased, so the overall consumption of systemic antibacterials (ATC code J01) decreased by approximately 10%; however, the reduction was not statistically significant. The incidence density and crude death rate of CR-Kp hospital-acquired infections at 28 days decreased, respectively, by 88.14% and by 88.85%. Although the intervention was very successful regarding its targets, it did not result in a decrease in total antimicrobial consumption, which is probably the hardest target to attain. One may comment that this study illustrates the “squeezing the balloon” effect, i.e., reduction in consumption of one or more antimicrobials results in increased consumption of others, so total consumption remains stable [9].

Spernovassilis et al. examined various quantitative and qualitative prescribing indicators before and after the implementation of a carbapenem-focused ASP, conducting two point prevalence surveys (PPSs) in a University Hospital in a health care setting with high AMR rates in October 2019 and October 2020 [10]. They found that there was a substantial decrease in the percentage of patients receiving carbapenems from 10.3% to 4.9%, without a concomitant increase in the use of most other antibiotics for multidrug-resistant Gram-negative bacteria, such as colistin and, most importantly, ceftolozane/tazobactam and ceftazidime/avibactam. It is important to note that the second PPS was conducted during the first COVID-19 wave, a period during which hospital admissions increased and inappropriate use of antimicrobials was often observed [11].

One of the most difficult problems for ASPs is inappropriate perioperative antimicrobial prophylaxis (PAP), because compliance with guidelines regarding the choice and, in particular, the duration of administration of antimicrobials is unacceptably low [12]. Kostourou et al. investigated the effect of a simple e-prescribing intervention in patients undergoing cardiac surgery, where the guidelines suggest that the duration of antimicrobial prophylaxis is up to 48 h postoperatively. The intervention was a reminder of the institutional PAP guidelines, which appeared when the prescribing surgeon selected “Prophylactic antimicrobial therapy” in the EMR menu. The primary outcome was adherence to the suggested duration of PAP, as this was the main area of non-compliance with the institutional guidelines. Adherence to the appropriate duration of PAP was very low before the intervention (4.0%) and it increased to 15.0% after the intervention; however, it was still inappropriately low. The authors suggest that despite the small effect size, such simple interventions might be useful as a complement to multimodal interventions and have the advantage of easy implementation and possibly better sustainability [13].

Among the populations of the hospitalized patients, Hematopoietic Stem Cell Transplant (HSCT) and Oncology patients represent a population with complex and unique problems for ASPs. Majumdar et al. review the challenges and opportunities in implementing ASPs for these patients, which are characterized by multiple risk factors for infection and for the presence of multi-drug-resistant organisms (MDRO). Therapeutic decisions in these patients are very difficult, especially regarding empirical antimicrobial treatment, as the treating physician must find a balance between the higher risk of poor outcomes and increased prevalence of MDROs in these patients on the one hand and the constant need for prudent use of antimicrobials on the other. Majumdar et al. provide a summary of the unique challenges and opportunities for unmet needs in these patient populations [14].

As stated before, ASP teams should ideally be multidisciplinary and require the support of microbiology and infectious diseases services. However, the availability of these services to support ASPs should not be taken for granted. Intensive Care Units (ICUs), due to the complex infectious disease challenges presented by their patients and the consequent high rate of antimicrobial use, are in need of ASPs. Catton et al. surveyed ICUs in the UK to assess the availability of microbiology, infection, AMS services, and antimicrobial prescribing practices [15]. They found prominent gaps, e.g., only 50% of ICUs had a dedicated infection control prevention nurse, antibiotic consumption data were not routinely discussed in a multi-disciplinary meeting, and local antibiotic surveillance data were available in only 47% of ICUs. This survey highlighted variations in practice and AMS services and might serve as a baseline to improve ASPs in the ICU.

## 3. Conclusions

It is not expected that these four research articles and the review from Majumdar et al. included in this Special Issue will cover fully, or even to a large extent, the multiple and diverse issues surrounding ASP in the General Hospital. However, they highlight important issues, such as carbapenem use and resistance [8,10], and focus on specific problems such as PAP in Cardiac Surgery [13] and special populations such as HSCT and Oncology patients [14]. In addition, they show that ASP requires support and collaborations, which are not always available [15]. In short, the included articles underscore why ASPs should be an essential practice in General Hospitals.
